# Retraction: Gravity-perfused airway-on-a-chip optimized for quantitative BSL-3 studies of SARS-CoV-2 infection: barrier permeability, cytokine production, immunohistochemistry, and viral load assays

**DOI:** 10.1039/d4lc90106a

**Published:** 2025-10-13

**Authors:** Shannon L. Faley, Niloufar A. Boghdeh, David K. Schaffer, Eric C. Spivey, Farhang Alem, Aarthi Narayanan, John P. Wikswo, Jacquelyn A. Brown

**Affiliations:** a Vanderbilt Institute for Integrative Biosystems Research and Education, Vanderbilt University Nashville TN 37235 USA; b Department of Biomedical Engineering, Vanderbilt University Nashville TN 37235 USA john.wikswo@vanderbilt.edu; c Biomedical Research Laboratory, Institute of Biohealth Innovation, George Mason University Manassas VA 20110 USA; d Department of Physics and Astronomy, Vanderbilt University Nashville TN 37235 USA; e College of Science, Department of Biology, George Mason University Fairfax VA 22030 USA; f Department of Molecular Physiology and Biophysics, Vanderbilt University Nashville TN 37232 USA

## Abstract

Retraction of ‘Gravity-perfused airway-on-a-chip optimized for quantitative BSL-3 studies of SARS-CoV-2 infection: barrier permeability, cytokine production, immunohistochemistry, and viral load assays’ by Shannon L. Faley *et al.*, *Lab Chip*, 2024, **24**, 1794–1807, https://doi.org/10.1039/D3LC00894K.

We, the authors, hereby wholly retract this article due to the discovery of significant errors including typographical labelling and nomenclature errors, figure errors and missing references.

Having consulted with independent experts, the Royal Society of Chemistry has determined the cumulative effect of all the errors and subsequent necessary corrections to be non-trivial, and therefore that the best course of action is retraction and republication of the article with the correct data. The Royal Society of Chemistry is happy that the overall conclusions of the paper are not affected by these errors, and therefore that republication of the work with the correct data is appropriate. The republished article was peer reviewed and can be found at https://doi.org/10.1039/D5LC00510H.

The authors are extremely grateful to the reader and the Royal Society of Chemistry for their assistance to correct the original article and agree with the decision to retract and republish this article with the corrected information.

Signed: Shannon L. Faley, Niloufar A. Boghdeh, David K. Schaffer, Eric C. Spivey, Farhang Alem, Aarthi Narayanan, John P. Wikswo and Jacquelyn A. Brown, 26th September 2025.

Retraction endorsed by Philippa Ross, Executive Editor, *Lab on a Chip.*

